# Efficiency of Sensory Substitution Devices Alone and in Combination With Self-Motion for Spatial Navigation in Sighted and Visually Impaired

**DOI:** 10.3389/fpsyg.2020.01443

**Published:** 2020-07-10

**Authors:** Crescent Jicol, Tayfun Lloyd-Esenkaya, Michael J. Proulx, Simon Lange-Smith, Meike Scheller, Eamonn O'Neill, Karin Petrini

**Affiliations:** ^1^ Department of Psychology, University of Bath, Bath, United Kingdom; ^2^ Department of Computer Science, University of Bath, Bath, United Kingdom; ^3^ School of Sport and Exercise Sciences, Liverpool John Moores University, Liverpool, United Kingdom

**Keywords:** navigation, visual impairment and blindness, sensory substitution device, audiotactile, spatial cognition, egocentric, allocentric, multisensory integration

## Abstract

Human adults can optimally combine vision with self-motion to facilitate navigation. In the absence of visual input (e.g., dark environments and visual impairments), sensory substitution devices (SSDs), such as The vOICe or BrainPort, which translate visual information into auditory or tactile information, could be used to increase navigation precision when integrated together or with self-motion. In Experiment 1, we compared and assessed together The vOICe and BrainPort in aerial maps task performed by a group of sighted participants. In Experiment 2, we examined whether sighted individuals and a group of visually impaired (VI) individuals could benefit from using The vOICe, with and without self-motion, to accurately navigate a three-dimensional (3D) environment. In both studies, 3D motion tracking data were used to determine the level of precision with which participants performed two different tasks (an egocentric and an allocentric task) and three different conditions (two unisensory conditions and one multisensory condition). In Experiment 1, we found no benefit of using the devices together. In Experiment 2, the sighted performance during The vOICe was almost as good as that for self-motion despite a short training period, although we found no benefit (reduction in variability) of using The vOICe and self-motion in combination compared to the two in isolation. In contrast, the group of VI participants did benefit from combining The vOICe and self-motion despite the low number of trials. Finally, while both groups became more accurate in their use of The vOICe with increased trials, only the VI group showed an increased level of accuracy in the combined condition. Our findings highlight how exploiting non-visual multisensory integration to develop new assistive technologies could be key to help blind and VI persons, especially due to their difficulty in attaining allocentric information.

## Introduction

Our world, built by the sighted for the sighted, poses significant challenges for the estimated 252 million visually impaired (VI) individuals worldwide (Bourne et al., 2017). Furthermore, visual impairments and blindness have been estimated to drastically increase in the next 30 years leading to approximately 4 million (when only considering the United Kingdom) living with sight loss (Future Sight Loss, 2009, pp. 43–44).

The eyes are our window to where we are and what is around us in the environment. Vision, with its higher spatial resolution, normally provides the most reliable information when it comes to spatial tasks in general, and to navigation specifically. Evidence that vision dominates other senses during spatial tasks comes from developmental studies. These studies show that children use visual information to calibrate (teach) other sensory cues during spatial tasks (e.g., Gori et al., 2008, 2010, 2012; Petrini et al., 2016) and also show that children have difficulties discounting or ignoring visual information even when it is irrelevant for the task (Innes-Brown et al., 2011; Downing et al., 2015; Petrini et al., 2015). In spatial navigation, vision is so relevant that it can influence even how humans find their way in the dark back to a previously seen location (Tcheang et al., 2011; Petrini et al., 2016). For example, a study using immersive virtual reality showed that, after being presented with conflicting visual information, adult sighted participants used a representation combining visual and self-motion cues to find their way back to the start in darkness (Tcheang et al., 2011).

However, when vision is absent or less reliable (e.g., in a poorly lit environment), our reliance on other sensory cues such as sound becomes essential. This holds especially true for blind individuals who need to mostly or completely rely on other sensory cues to perform daily tasks (e.g., locating a person by his/her voice). Navigation is a particularly important but demanding task for blind individuals as they not only have to find their way by using less reliable spatial information but they also have to avoid collision with a huge number and variety of obstacles in the environment (e.g., objects, people, and animals). Several studies have demonstrated how visual experience is essential for the typical development of spatial cognition and navigation abilities (Pasqualotto and Proulx, 2012). This, however, is not true for all kinds of navigation but it seems to be specific to navigation tasks that require an allocentric (i.e., a spatial representation built on the relative position of objects in the environment), rather than an egocentric (i.e., a spatial representation built on the subject’s own position in the environment), representation of space (Pasqualotto et al., 2013; Iachini et al., 2014). For example, Iachini et al. (2014) reported that congenitally blind participants, when compared to late blind and sighted participants, found it difficult to represent spatial information allocentrically, but not egocentrically, during a large-scale space navigation task. Accumulating evidence of this type has prompted the development of numerous types of technological aids aimed to help individuals with visual deficits during navigation requiring allocentric representation (i.e., in large-scale environments).

Among these technological approaches, sensory substitution devices (SSDs) have received a great deal of interest in the last few decades. SSDs are noninvasive technologies that exploit the ability of the brain to adapt and to process the lost sensory information (vision) through the other unaffected senses (e.g., “seeing through the ears”; Bach-y-Rita et al., 1969; Meijer, 1992). SSDs are not only noninvasive and much cheaper than other alternatives (e.g., sensory restoration devices) but are also better suited for use with different types of visual deficits, including congenital blindness. This is because they do not require a developed visual system and/or any previous visual knowledge (Proulx et al., 2014a).

A freely available SSD is The vOICe, which uses an image-to-sound conversion algorithm which receives input from a camera and transposes it into 1-s auditory “soundscapes” (Meijer, 1992). The vOICe algorithm transforms visual images by scanning them from left-to-right, converting them into grayscale, and subdividing them into pixels. Each pixel is then converted into sound (or “sonified”) based on its luminance, horizontal position, and vertical position. High luminance pixels sound louder than low luminance pixels, pixels on the left of the visual field are played before those on the right, and pixels at the top have a higher pitch than those at the bottom (Meijer, 1992).

The vOICe has been demonstrated to allow VI individuals to access visual information through audition, allowing object recognition and localization (Auvray et al., 2007). However, The vOICe is limited as users find it difficult to distinguish between multiple objects which are vertically aligned, as it is difficult to distinguish between the pitches of sounds which are played simultaneously (Brown et al., 2015). Similarly, due to the nature of the left-to-right scanning that creates soundscapes, it is difficult to process horizontally aligned objects simultaneously, as their respective sounds are played at different junctures. Nevertheless, the benefit of The vOICe cannot be understated, as it confers superior spatial resolution to all the other tactile-visual sensory substitution systems (e.g., BrainPort; for details see Bach-y-Rita and Kercel, 2003; Haigh et al., 2013; Proulx et al., 2014a).

An alternative SSD is the BrainPort, a visual-to-tactile aid. This device operates by transforming images into a pattern of electrical stimulation delivered *via* an electrode array that sits atop the tongue (Bach-y-Rita and Kercel, 2003). The device is used by exploring this electrode pad, thus objects can be processed theoretically in parallel (Arditi and Tian, 2013), and users might have no difficulty in distinguishing between vertically aligned objects. In addition, the BrainPort confers a superior temporal resolution to The vOICe, although its spatial resolution is inferior (Bach-y-Rita and Kercel, 2003).

That The vOICe and BrainPort each seem to have strengths where the other has weaknesses raises the question of whether the unaffected sensorimotor ability (e.g., self-motion) could be integrated with one or even both of these simultaneously during spatial navigation. Optimal concurrent use of two or more SSDs would be reliant on multisensory integration, the process by which information from different senses is combined to form a holistic percept (Stein et al., 2009). Thus, concurrent use of multiple SSDs could allow multisensory integration of incoming information, whereby the advantages of each device compensates for the respective limitations of the other (Shull and Damian, 2015). Or the use of these devices concurrently with another sensorimotor ability could increase precision and accuracy during spatial navigation by integrating these multiple information sources in absence of vision.

The ability to use a multimodal representation of space in blind individuals when navigating their environment, however, has not received support in persons with restored vision through a retinal prosthesis. Garcia et al. (2015) examined the ability of a group of adult patients with ARGUS II retinal prosthesis to use the restored visual information to navigate a simple two-legged path. The patients, an age-matched control group and another younger control group, had to retrace a two-legged path (two sides of a triangle they previously experienced) in one task and go back to the start point after walking the same two-legged path in another task (i.e., they had to complete the triangle by walking as precisely as possible the remaining third side). Before reproducing the path or completing the triangle, participants could walk (by being guided) the two-legged path with either an indirect visual landmark or no visual landmark. Garcia et al. (2015) showed that, in contrast to sighted individuals, these patients did not use a combined representation of visual and self-motion cues when navigating (when reproducing the path or completing the triangle) but relied entirely on self-motion (Garcia et al., 2015). Thus, it appears that a multimodal representation of space (a single and coherent representation of space obtained by integrating the restored visual information with self-motion) was not formed in these blind individuals.

This stands in contrast to existing evidence from neuroscience, which suggests that congenitally blind individuals can recruit visual areas when recognizing sounds, shapes, and movements through SSDs (De Volder et al., 1999; Poirier et al., 2007), in addition to areas, such as parahippocampus and visual cortex, that are essential for successful spatial navigation in sighted individuals (Kupers et al., 2010). A possible explanation is that blind individuals may usually form a non-visual multimodal representation of space with the unaffected sensory information (e.g., sound and self-motion). In that case, using the restored visual information would be detrimental rather than helpful as the possible representation of space with the restored visual information (with a far lower resolution than typical vision) is poorer than a non-visual multimodal representation of space. Consequently, forming a multisensory representation of space and benefitting from it could be possible for VI and blind individuals when using non-visual information as provided by the SSDs. That blind and VI individuals may use a non-visual multisensory representation of space to increase their accuracy and precision is supported by recent findings showing that an audiotactile map (delivered through a touchpad) was more efficient than either a tactile only map or only walking during a navigation task (Papadopoulos et al., 2018).

The ability of blind/VI and sighted blindfolded individuals to use SSDs (Chebat et al., 2011, 2015; Maidenbaum et al., 2014; Kolarik et al., 2017) efficiently during spatial navigation, even after a short training, is well-known. For example, Chebat et al. (2011) showed that congenitally blind participants had an enhanced ability to detect and avoid obstacles compared to blindfolded sighted when using a tongue display unit (TDU), and Chebat et al. (2015) showed that congenitally blind, low vision, and late blind individuals could achieve the sighted (non-blindfolded) performance in a real and virtual maze after few trials with the EyeCane (a device that uses sound and vibration to deliver information about distances). Chebat et al. (2015) also showed that participants could improve their spatial perception and form a cognitive map through the learning experience afforded by the EyeCane. However, what remains unclear is whether the formation of a cognitive map combining non-visual information can speed up learning and provide better precision and accuracy to VI and blind users. Understanding whether the integration of different non-visual cues can improve VI spatial navigation has both important theoretical and applicative significance. On the one hand, it has important implications for the development, training, and application of existent and new aids for the blinds. On the other hand, it could bring support to a convergent model of spatial learning (Schinazi et al., 2016) in the blind and VI, by showing that even when using less effective cues for navigation, blind and VI can learn to perform as well as sighted by increasing their precision through non-visual multisensory integration.

Here, we examine this possibility by first testing whether combining a vision-to-sound and a vision-to-tactile information as provided by two SSDs can enhance navigation performance in a group of blind-folded sighted participants. Next, we tested whether combining the information from one SSD with existing and unaffected senses (e.g., self-motion and proprioception) can improve navigation precision and accuracy in a group of blind-folded sighted participants and a group of VI individuals. To test the formation of a cognitive map, we asked participants to perform the navigation task (walking to a target location) in darkness after experiencing the environment under different conditions (e.g., with an SSD or with self-motion). To test whether there was an increase in accuracy and precision (when combining either information from different SSDs or from one device and the available self-motion information), we used a maximum likelihood estimation (MLE) framework (i.e., we compared the reduction in variability for the measured combined condition to that obtained for each sense separately and to the reduction in variability predicted by the MLE; Ernst and Banks, 2002). Under the MLE framework, we expect to see a significant reduction in performance variance (or reduced uncertainty) as predicted by the model when the variance for the unimodal conditions (e.g., when using the two SSDs in isolation) are similar, or in other words when the reliability of the cues to be integrated are similar. Hence, the tasks used here were chosen to be fairly easy and straightforward to assure that a similar level of performance with different devices could be achieved.

In Experiment 1, we examine whether a non-visual multisensory representation of space can improve the navigation performance of a group of sighted blindfolded individuals when using a tactile or auditory SSD (i.e., The vOICe or the BrainPort) or the two together (The vOICe and BrainPort) in an egocentric and allocentric aerial map task. Aerial maps are the most common representations provided to people for building layouts and cities, and blind persons have been shown to benefit from a tactile aerial representation when navigating an unfamiliar environment (Espinosa et al., 1998), probably because it removes the lack of depth perception as a barrier for VI individuals. Furthermore, a survey representation which encodes external and unfamiliar information of the environment (like in an aerial or map-like view) is more severely affected by lack of vision when compared to route (serial)-based representation (Tinti et al., 2006). Hence, we used an aerial map task to assess the efficiency of different SSDs alone or in combination. We chose this task also based on recent evidence that the use of audiotactile maps to build cognitive spatial representations are more efficient than using only a tactile map or walking in an unfamiliar environment (Papadopoulos et al., 2018). We hypothesized an improved performance (reduced variance) on a distance estimation-based navigation task when participants explored aerial maps using The vOICe and BrainPort together than when using either of these devices in isolation. We also hypothesized an increase in accuracy with a number of trials for all the conditions.

In Experiment 2, we examine whether a non-visual multisensory representation of space can improve the navigation performance of a group of sighted and a group of VI blindfolded individuals when using self-motion or The vOICe or the two together in an egocentric and allocentric spatial navigation task. We hypothesized an improved performance (reduced variance) on the navigation task using The vOICe and self-motion together than when using either The vOICe or self-motion in isolation, especially for the VI group. We also hypothesized an increase in accuracy with a number of trials for all the conditions, especially for the VI group.

## Experiment 1

### Method

#### Participants

Thirty students (15 males and 15 females), aged 18–22 (*M* = 20.38, *SD* = 0.924), from the University of Bath, UK, participated in the experiment. Due to technical problems, some of the trials for three participants were not saved correctly and thus we had to exclude these participants. Hence, the data for twenty-seven participants were included in the analysis. Twenty-five were self-reportedly right-handed. All participants had normal vision and audition and were naïve to The vOICe, BrainPort, and the laboratory where the experiment took place. Participants were reimbursed £5 for their time. All participants provided informed consent and were debriefed. The experiment was approved by the University of Bath Psychology Department Ethics Committee (Ethics Code 16:180).

#### Apparatus

The experiment took place in an 11 m × 7 m laboratory. Two configurations of four target points (each 50 cm × 50 cm) were marked on the floor of the laboratory (see Supplementary Figures S1, S2), one for training and one for the experimental procedure. These configurations were based on studies by Garcia et al. (2015) and Petrini et al. (2016).

**Figure 1:**
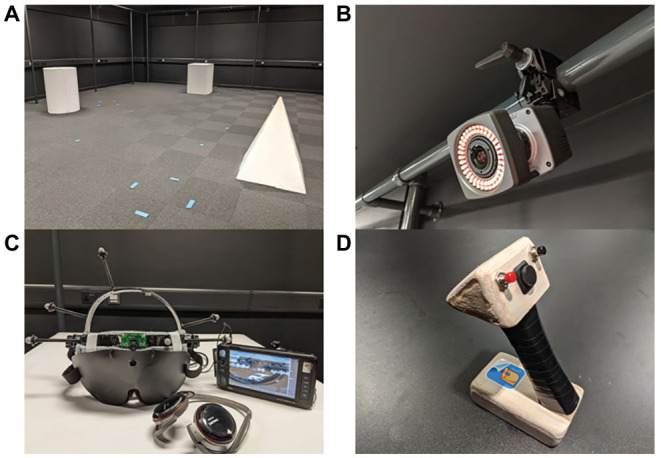
**(A)** The arrangement of the three objects in the Virtual Reality (VR) Lab as viewed from the start point. **(B)** A Vicon Bonita infrared camera is shown. A system comprising of eight identical cameras was used to obtain three-dimensional (3D) coordinates of the participants’ head location. **(C)** The helmet that was constructed for the task; incorporating the blindfold, camera, and trackers. The helmet is wired to a mini PC which runs The vOICe algorithm and plays back the soundscapes through a pair of headphones. **(D)** The remote that was constructed to trigger, stop, and forward trials of tracking from the Vicon tracking system.

The laboratory was equipped to record motion tracking data, using a Vicon Bonita system consisting of eight infrared cameras (see Figure 1B), which tracked five reflectors on the motion tracking helmet, to which a blindfold was attached (see Figure 1C). The Vicon system was controlled through a Python 3.0 script using Vizard libraries. A remote for controlling the script was used to control tracking for each navigation trial (see Figure 1D).

The BrainPort device consists of three parts: camera glasses, the processor unit, and the Intra-Oral Device (IOD). A laptop connected the BrainPort’s software (vRemote) to the live feed from the camera glasses to display the settings and allow correct positioning of the stimuli. Auditory stimuli were played from the same laptop *via* Philips stereo headphones. The headphones we used were open in the sense that participants could still hear sounds in the room to some extent, as well as their own footsteps. This was done so as to replicate as closely possible to a real environment which will have noises (information normally used by the blind and VI). These noises were always kept constant though throughout the conditions of the study so as not to add a confounding variable. Previous literature suggests that a head-mounted camera performs better than a hand-held camera while using The vOICe for navigation purposes (Brown et al., 2011). As a result, we designed a helmet with a blindfold (Mindfold Eye Mask) and reflectors used for motion tracking attached. A USB camera (ELP 480P webcam with 120° view) was mounted to the middle of the blindfold (see Figure 1C). The USB webcam was connected to a mini-PC (1.3 Ghz Intel Atom processor, 1 GB RAM) running Windows XP and The vOICe (Meijer, 1992). Participants used Philips SHS 5200 neckband headphones to listen to the soundscapes.

We used the default settings of The vOICe algorithm aside from changing the zoom to 2×. This enabled participants to observe the objects separately, group them two by two or explore them all at the same time. The experiment took place in the Virtual Reality (VR) Lab (11 m × 7 m). The three-dimensional (3D) objects developed for the study were a cylinder, a cube, and a four-faced pyramid of the same height (60 cm; see Figure 1A). We used different shapes intentionally as we wanted the soundscapes returned by The vOICe to be different so as to replicate more closely real environments where various objects are available. However, the three objects had similar dimensions as they had the same width and length.

### Materials

#### Experimental Stimulus Design

Aerial perspectives of the training and experimental point configurations marked on the laboratory floor were digitally recreated to scale using AutoCad (Version 21.0, AutoDesk, Inc., Mill Valley, California, United States). These were the “aerial maps,” with each target and the start point being indicated by a white square on a black background (see Supplementary Figure S2). All stimuli were transformed into soundscapes using The vOICe’s image sonification algorithm (Meijer, 1992) at the following settings: 2-s scan rate, normal contrast, and foveal view off. A5 sized prints of all stimuli were placed in front of the BrainPort camera and were explored *via* the IOD at the following settings: zoom 33°, invert off, contrast high, lighting low, tilt 25°, and lock off. This ensured that the visual information being transformed by both devices was congruent to ensure that multisensory integration was not prevented (Schinazi et al., 2016).

#### Training Stimulus Design

The training stimuli consisted of a set of four lines and five sets of circles (all white on a black background), which occupied approximately the same visual area (see Supplementary Figure S3). The stimuli were produced in the same fashion and using the same settings as the experimental stimuli.

#### Conditions

The conditions of the experimental procedure comprised of two unimodal conditions: The vOICe only (vOICe) and BrainPort only (TDU), and one bimodal condition: The vOICe plus BrainPort (vOICeTDU). In each condition, the same aerial map was delivered, and 10 wayfinding task-pairs were completed. Thus, in total, every participant completed 60 wayfinding tasks, based on the same target configuration. The order of wayfinding tasks was counterbalanced among trials and conditions. This was done to minimize a potential confound of participants learning the configuration of target points over subsequent conditions.

#### Navigation Tasks

**Figure 2:**
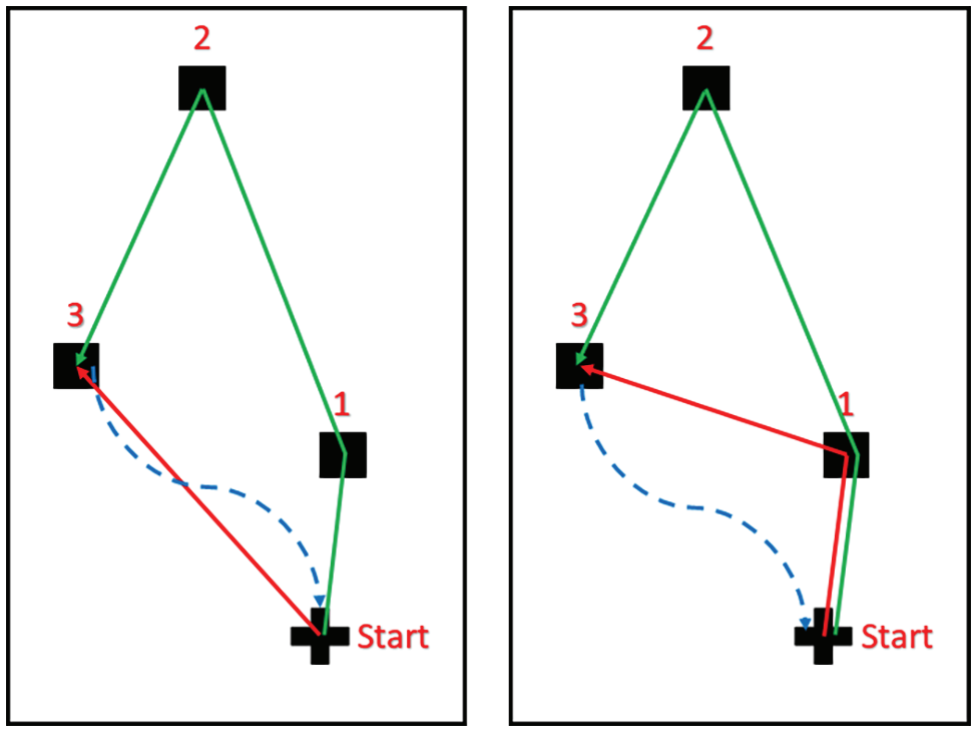
The egocentric (**left** panel) and allocentric (**right** panel) navigation tasks. Objects are represented by the black squares and the cross represents the starting point of the navigation trial. The solid green lines represent the encoding phase in which participants were guided starting from the start point to Object 1, then Object 2, and finally to Object 3. The dashed blue lines represent the scrambled routes used to take participants back to the start point after the encoding phase (green line). The solid red lines represent the routes participants had to navigate in darkness following the encoding phase (red continuous lines).

Each wayfinding task-pair comprised of an egocentric task and an allocentric task. In the egocentric task, participants navigated directly to target 3 from the start point (Figure 2). In the allocentric task, participants navigated from the start point to target 1 and then to target 3 (Figure 2). The experimenter oriented participants toward their first target they were to navigate to prior to commencing each task.

Participants’ motion during the wayfinding tasks was tracked: commencing once they were ready to begin each task and terminating once they announced that they had reached the target location. They were then returned to the start point *via* an indirect route to discourage them from trying to estimate the distance between their final position and the start point from the route the experimenter took them rather than the SSD(s).

#### Procedure

The study consisted of three phases: basic training, active training, and the experimental procedure. Prior to the study, the experimenter collected demographic information from the participants (age, handedness, and gender). They were then blindfolded to prevent viewing the interior of the laboratory.

#### Basic Training

Upon beginning the study, participants were trained to use the two SSDs. This procedure utilized the training stimuli. The device that participants were trained with first was counterbalanced in an ABAB fashion. First, the experimenter would explain the mechanisms of action of both SSDs. Then, for each training stimulus, the experimenter either played the auditory file for The vOICe or placed the relevant printed stimulus in front of the BrainPort camera, for 10 s. Participants were asked to use the relevant device to identify and count the lines or circles that were presented to them. The question was left open-ended, so the likelihood of participants correctly identifying the stimulus by chance was negligible. If the stimulus was identified, training would progress to the next stimulus. If not, feedback was provided, and the mechanism of action of each SSD was explained again. This process continued until participants were able to identify and count all training stimuli.

#### Active Training

The purpose of the active training was to give participants a sense of the scale, how the distances between the target points they experienced using the SSDs equated to physical distances. The active training mirrored the three experimental conditions in terms of the utilized exploration methods (vOICe, TDU, or vOICeTDU) and was counterbalanced mirroring the experimental procedure.

This procedure utilized the aerial map of the training target configuration (Supplementary Figure S2), which was delivered *via* the SSDs. Participants were instructed to explore the training aerial map *via* the SSD(s) for as long as required to identify and localize all points. Before each practice trial, participants were told that they would be taken to the starting point and oriented in the direction in which they would need to move initially (depending on whether they were doing an egocentric or an allocentric task). They were then told to walk as far as they needed and turn as much as they needed to reach the target point. During this practice phase, participants received feedback, that is, if they made a mistake in estimating distance or angle then the experimenter would correct them and tell them whether they had over/underestimated. This was done at each target location and for both distance and rotation, and thus, for the allocentric task, participants received feedback after the first (Object 1, see Figure 2 right panel) and second target (Object 3, see Figure 2 right panel), while for the egocentric task feedback was received for the only target used for the task (Object 3, see Figure 2 left panel). They would then complete two trials of the navigation task, one allocentric and one egocentric with the order counterbalanced. At the end of active training, participants were led outside the laboratory for a 5-min break.

#### Experimental Procedure

Each experimental condition was identical, the only difference being the SSD the participants used to explore the aerial map. This procedure utilized the experimental target configuration and respective aerial map (Figure 2 and Supplementary Figure S2). Upon beginning a condition, participants used the device(s) specified by the condition to explore the aerial map for 10 s (this was an arbitrary time limit enforced to standardize stimulus exposure). That is, participants used the different devices (depending on the condition at hand) to scan the room before attempting the navigation task, while during the navigation task only self-motion was used. When using both devices together, alignment between the two signals was controlled by the participant by activating the BrainPort as soon as The vOICe information started, so that the two devices started to deliver information at approximately the same time. The decision to let the participants control for the start of the BrainPort was taken to better approximate a real condition in which the user would have control on what device to use and when. They would then complete two trials of the navigation task, one allocentric and one egocentric with the order counterbalanced, using self-motion. Upon completing both trials, participants were led back to the SSD apparatus, and they used the device(s) for the given condition for another 10 s, and then completed another pair of navigation trials. This process was repeated until 10 pairs of navigation trials were completed. Once a condition was completed, participants were led outside the laboratory and had another break. The process was then repeated for the remaining two conditions. Once participants had completed the navigation tasks, they were taken outside the laboratory and debriefed, gave final consent, and were paid, thus concluding the experiment.

### Results

#### Individual Estimates

**Figure 3:**
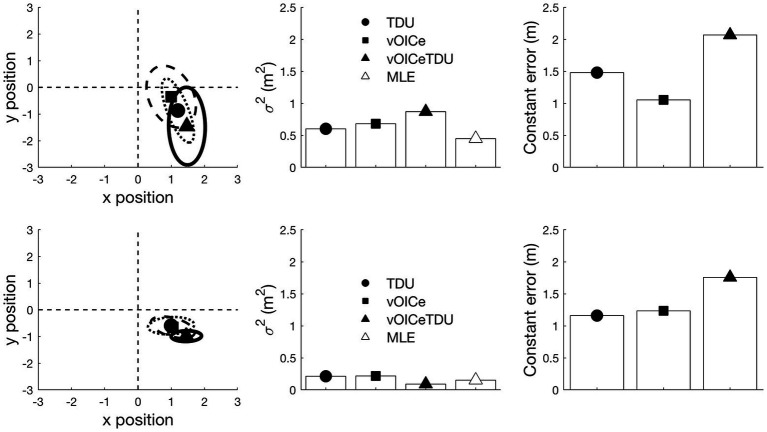
The locations at which the participant stopped relative to the true target position (0,0 in the **left panel**) was used to derive the individual variable error (the area of the ellipse) and constant error (the distance from the center of the ellipse to the target location) for each task (allocentric and egocentric) and each condition (vOICe, TDU, and vOICeTDU) separately. The unit for the x and y position is in meters. Individual estimates for vOICe, tongue display unit (TDU), and vOICeTDU conditions for the allocentric task are shown in the top panel, while for egocentric task, the estimates are shown in the bottom panel for the same participant. The triangle labeled maximum likelihood estimation (MLE) (white) in the central panels refer to the variability predicted by the MLE model for the combined condition (vOICeTDU). The predicted estimate (MLE) was calculated for each subject, and then averaged, by entering the individual vOICe ($\sigma_{vOICe}$) and TDU ($\sigma_{TDU}$) measure of variable error into the equation $\frac{\sigma_{vOICeTDU=}^{2}\;\;\;\;\;\sigma_{vOICe}^{2}\sigma_{TDU}^{2}}{\;\sigma_{vOICe}^{2}+\sigma_{TDU}^{2}}$.

The tracked coordinates obtained through the Vicon system were processed using MATLAB (Version R2018b, The MathWorks, Inc.) and Psychtoolbox command Library (Brainard, 1997; Pelli, 1997). For each participant’s end positions (when the participant decided he/she arrived at the object’s target position), a bivariate normal distribution was fitted (Figure 3), which enabled the estimation of x mean, y mean, x variance, and y variance. The FASTCMD algorithm (Rousseeuw and Driessen, 1999), as implemented by the MATLAB Libra toolbox (Verboven and Hubert, 2005), was used for a robust estimation of these values, with the assumption of 1% aberrant (outlier) values (i.e., a value of 0.99 for the alpha parameter). For each participant, a single variable error was computed by using the sum of the variance of x and y directions of the fitted bivariate distribution (black ellipses in Figure 3). Secondly, a measure of constant error was calculated as the distance between the center of the fitted bivariate distribution (center of the black ellipses in Figure 3) and the correct position for the target object (Object 3). Variable error is expected to reduce when participants are able to combine multiple modalities and in line with the MLE model (Ernst and Banks, 2002; Alais and Carlile, 2005; Cheng et al., 2007; Van der Burg et al., 2015; Noel et al., 2016). On the other hand, constant error represents a systematic navigational bias. That is, it reoccurs over multiple trials and is consistent. Constant error is expected to reduce when less biased information is available.

#### Group Analysis

The variable error estimates (obtained as size of the individual ellipsis for each condition, see Figure 3) and the constant error estimates (obtained as the distance of the center of each individual ellipsis from the correct target position, point 0,0 in Figure 3) were tested to determine whether they were normally distributed. As the majority of conditions did not meet the assumption of normal distribution (Shapiro-Wilk, *p* < 0.05), we used Wilcoxon tests to examine differences between conditions (e.g., vOICeTDU vs. vOICe) within each group, and Mann Whitney U tests to compare the two groups’ performances in each condition. We then used Pearson’s correlation analyses (as assumption of linearity was met) to determine whether the number of trials (from 1 to 10) was associated with changes in constant error (i.e., accuracy), in other words, whether there was a decrease in error (or increase in accuracy) with increased number of trials. For directional hypotheses, the reported results are one-tailed.

**Figure 4:**
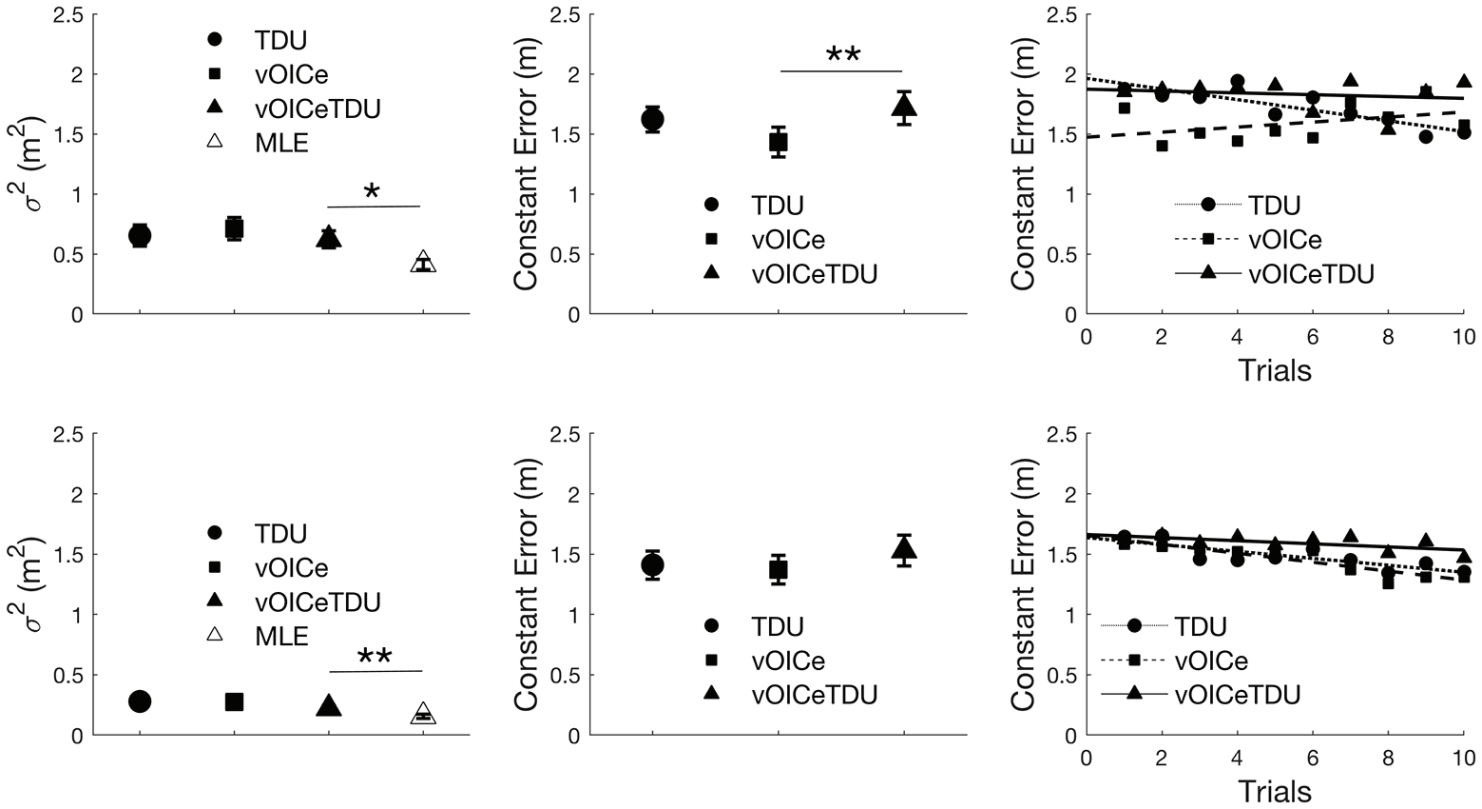
Average variable error (**left panel**), constant error (**middle panel**), and relation between average constant error and number of trials (**right panels**) for the allocentric (**top panels**) and egocentric (**bottom panels**) task. vOICe = vOICe condition alone; TDU = BrainPort alone; vOICeTDU = vOICe + TDU. The marker labeled MLE (in white) in the left panel refers to the reduction in variability predicted by the MLE model. How MLE was calculated is explained in Figure 3 caption. Please see Supplementary Figure S4 for boxplot with median and interquartile range (IQR) measures. ^**^ *p* < 0.01; ^*^ *p* < 0.05. Error bars represent the standard error of the mean.

Figure 4 (left panels) shows the results for the variable error in the allocentric (top panels) and egocentric (bottom panels) tasks. Wilcoxon tests were used to compare the variable error between the bimodal (vOICeTDU) and the unimodal conditions (vOICe and TDU) and between the measured bimodal (vOICeTDU) and the predicted bimodal (MLE) conditions separately for the allocentric and egocentric tasks. The analysis showed no significant difference between vOICeTDU and the unimodal (vOICe and TDU) conditions for both tasks, *Z* ≤ −0.953, *p* ≥ 0.170, one-tailed. There was, however, a significant difference between vOICeTDU and MLE for both tasks (*Z* ≥ −2.463, *p* ≤ 0.014) indicating that the level of variability for the bimodal condition was not accurately predicted by the MLE model.

A similar analysis was performed on the constant error measures (Figure 4 middle panels), and it showed no significant difference between vOICeTDU and TDU for both egocentric and allocentric task (*Z* ≤ −1.410, *p* ≥ 0.079, one-tailed) and a significant difference between vOICeTDU and vOICe in the allocentric task (*Z* = −2.440, *p* = 0.007, one-tailed), indicating higher accuracy and less bias with The vOICe alone, but only a trend in the egocentric task (*Z* = −1.600, *p* = 0.055, one-tailed).

Finally, we examined whether sighted participants showed any learning effect across the 10 trials within each sensory condition (vOICe, TDU, and vOICeTDU) for allocentric and egocentric task separately. Thus, Pearson correlations (given the data linearity) were used to analyze whether the average constant error decreased with an increase in number of trials, i.e., whether participants’ accuracy increased with practice. For the allocentric task, as shown in Figure 4 top right panel, a significant association between decrease in error and increase in trial number was found for the TDU condition (*r* = −0.863, *p* < 0.001, and one-tailed) but not for the vOICeTDU (*r* = −0.182, *p* = 0.308, and one-tailed) and vOICe condition (*r* = 0.424, *p* = 0.111, and one-tailed). In addition, vOICeTDU accuracy performance as a function of trials did not correlate with either the performance in The vOICe or TDU alone (*r* ≤ 0.039, *p* ≥ 0.458, and one-tailed). For the egocentric task, as shown in Figure 4 bottom right panel, a significant association between decrease in error and increase in trial number was found for the TDU condition (*r* = −0.795, *p* = 0.003, and one-tailed) and for The vOICe condition (*r* = −0.881, *p* < 0.001, and one-tailed), but not for the vOICeTDU condition (*r* = −0.499, *p* = 0.071, and one-tailed), although the combined condition did show a trend in this direction. Finally, vOICeTDU accuracy performance as a function of trials significantly correlated with both the performance in The vOICe or TDU alone (*r* ≥ 0.594, *p* ≤ 0.017, and one-tailed). This suggested that in the egocentric task the changes in accuracy in the bimodal condition (vOICeTDU) was driven by changes in accuracy for both The vOICe and TDU condition alone.

## Experiment 2

### Method

#### Participants

Experiment 2 had 32 sighted right-handed participants take part, with equal numbers of males (16, mean age of 23.4 and *SD* = 5.17) and females (16, mean age of 23.1 and *SD* = 2.70). Six VI participants were also recruited for the study (see Table 1 for participant details). All participants had normal hearing and sighted participants had normal or corrected-to-normal vision as assessed by self-report. They had no prior experience of The vOICe and never been to the VR Lab where the experiment took place. Participants were paid £20 for their time. Ethics approval was granted by the Psychology Research Ethics Committee of University of Bath (ethics reference 16-180).

**Table 1:** Clinical and demographic information for blind and low vision child participants.

Participant	Sex	Age	Age of onset	Diagnosis	Visual acuity (right eye; left eye) [logMAR]	Vision status
VI1	Female	18	Birth	Bilateral retinoblastoma, cataract, right enucleation	R -; L = 2.8	Congenitally blind
VI2	Male	21	Birth	Congenital bilateral cataracts (until 9 years), glaucoma, retinal detachment		Congenitally blind/sight restored
VI3	Female	18	6 years	Retinitis pigmentosa	R > 1.8; L > 1.8	Early blind
VI4	Male	61	11 years	Stargardt disease	R = 2.8; L = 2.8	Late blind
VI5	Female	20	12 years	Stargardt disease	L = 0.8; R = 0.8	Low vision
VI6	Female	49	41 years	Pathological myopia, choroidal neovascularization	R = 1.1; L = 0.8	Low vision

#### Apparatus

The apparatus was the same of that in Experiment 1; however, here only The vOICe was used.

#### Conditions

Three different conditions were utilized, The vOICe only (vOICe), self-motion only (SMO), and vOICe and self-motion combined (vOICeSMO). For the 32 sighted participants, we counterbalanced the tasks order, so that only 11 participants performed the combined vOICeSMO condition at the end. Since the aim of the study was to examine whether sighted and VI participants could use The vOICe when navigating to a target and whether they could benefit from using The vOICe with self-motion together, we tested the six VI participants with a task order for which the combined vOICeSMO condition was always at the end. This was necessary to allow for familiarization with The vOICe and self-motion tasks alone before testing for their combination. Hence, we compared the performance of the six VI to that of the 11 sighted that also had the combined vOICeSMO condition at the end. The data for all 32 sighted are presented as a reference for the 11 sighted to show if any difference emerged due to differences in task order.

Every condition defines the way participants were allowed to explore the objects and learn their physical locations. In vOICe, they stood at a marked location (the start point) and scanned the room and the objects with The vOICe but without moving. In SMO, participants were guided to each object in order (from Object 1 to 2 and then 3) while The vOICe was muted, and brought back to the start point *via* a scrambled path (Garcia et al., 2015; Petrini et al., 2016). In vOICeSMO, participants were guided to each object in the same order as SMO while The vOICe was on and brought back to the start point *via* a scrambled path (see Figure 2).

#### Navigation Tasks

We had two main navigation tasks: an egocentric navigation task, in which participants were asked to directly walk to Object 3 from the start point, and an allocentric navigation task, in which participants were asked to walk to Object 3 through the position of Object 1 (see Figure 2). For all conditions, the path to Object 3 either directly or indirectly was unfamiliar as during the encoding phase they were guided through the path formed by all three objects. Participants’ motion during the navigation tasks was tracked: motion tracking started once the participant was ready to start either the egocentric or allocentric task and stopped once they announced that they reached the target location. They were then guided back to the start point from where they stopped *via* a scrambled path and this motion was not recorded (Figure 2). Every time they explored the objects in a given condition, the objects were then removed from their locations before participants completed the navigation tasks. The order of the tasks (allocentric or egocentric) was counterbalanced after each trial and each participant completed 60 navigation tasks in total during the experimental phase (10 for egocentric task and 10 for allocentric task for vOICeSMO, vOICe, and SMO). For example, during a vOICeSMO trial, participants would be guided from Object 1 to Object 2 to Object 3 with both self-motion and The vOICe on during the encoding phase, then they would be guided back to the start point following a scramble path from Object 3, and finally the objects were removed and the testing phase would start. During the testing phase participants would be asked to either walk to Object 3 directly from their start position (egocentric task) or to walk to Object 3 through Object 1 position (allocentric task).

#### Experimental Procedure

Participants were initially welcomed to the Crossmodal Cognition Lab, where after reading an information slip and signing the consent form, they completed a brief demographics survey. Later, they observed an online presentation about the main principles of The vOICe algorithm with examples of simple and complex shapes and their sonifications. The third phase of the theoretical training consisted of a quiz containing 10 questions: each question had one soundscape and four multiple choice 2D simple shapes such as a white triangle on a black background. Participants were asked to pair the soundscape with the correct image. After each question, they were given a brief feedback on whether they had correctly answered the question. Immediately after the quiz, participants were introduced to the tracking helmet. This pre-experimental phase took around 30 min. Participants were then accompanied to the VR Lab, and were asked to wear the tracking helmet with the blindfold just before entering. The practical training to familiarize participants with using The vOICe in localization tasks involved 2 phases: room and object exploration and navigation training.

The practical training began with an accommodation phase where the three objects were placed in different locations on the floor and participants were guided through the room to explore the three objects and the room with The vOICe. Participants were further informed on how the soundscapes change with respect to the location of the object – i.e., while getting closer to or away from an object. This part of the practical training took around 15 min.

The navigation training mirrored the three experimental conditions in terms of the utilized exploration methods (vOICeSMO, vOICe, and SMO). However, the three objects and the start point were placed at different locations than in the main experiment. The order of the conditions during the practice for a specific participant was the same as in the actual experiment. This part of the training took around 30 min.

#### Main Experiment

During the main experiment, each object encoding condition (vOICeSMO, vOICe, and SMO) was followed by either the egocentric or allocentric task during which participants navigated to the object target with only the self-motion information. Five-min breaks were offered after each condition, in which participants were taken out of the lab and allowed to take off the helmet. The main experiment took approximately 1 h and 30 min.

### Results

#### Individual Estimates

**Figure 5:**
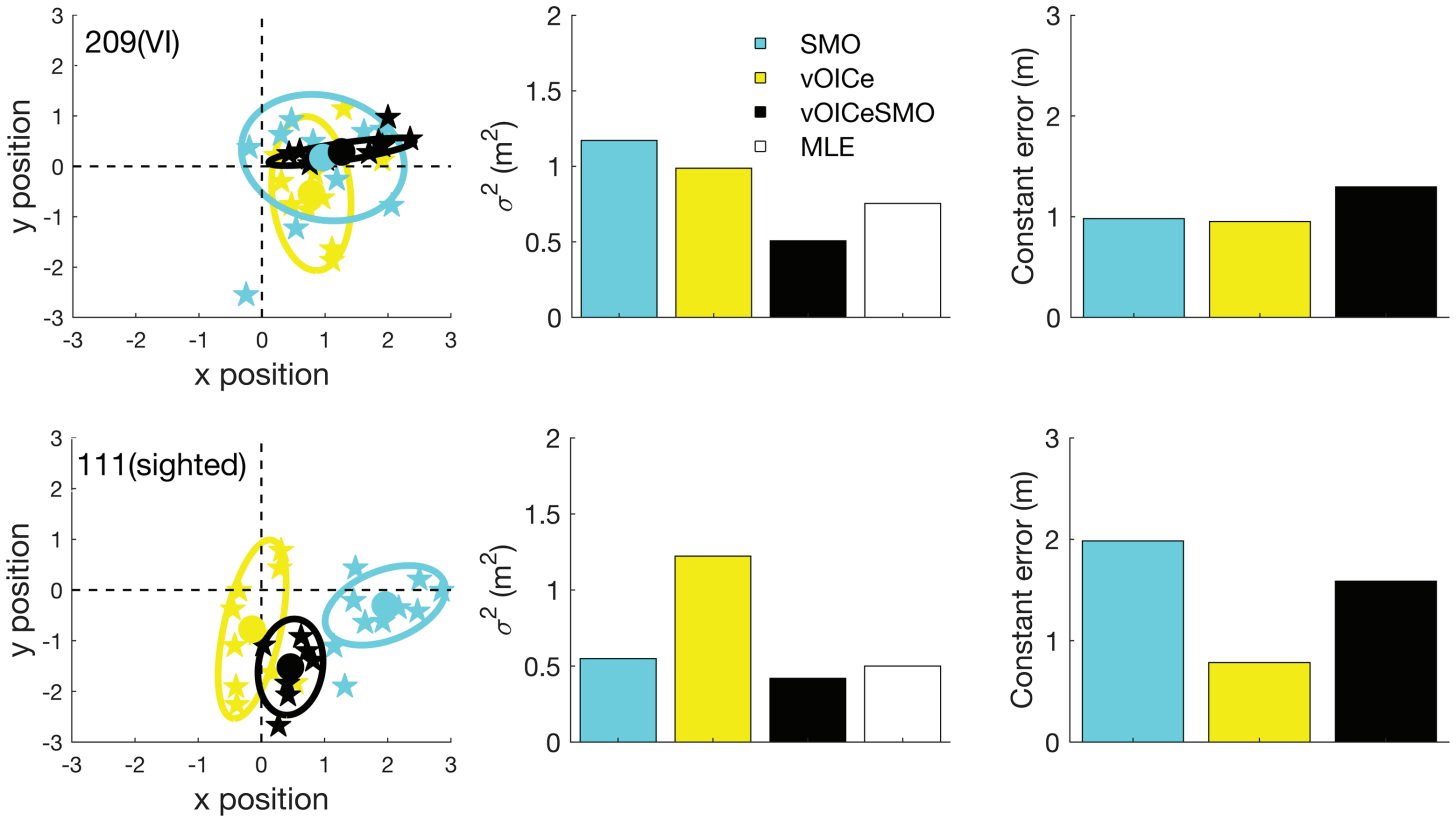
The locations at which the participant stopped relative to the true target position (0,0 in the **left panel**) was used to derive the individual variable error (the area of the ellipse) and constant error (the distance from the center of the ellipse to the target location) for each task (allocentric and egocentric) and each condition (vOICe, SMO, and vOICeSMO) separately. The unit for the x and y position is in meters. Individual estimates for self-motion (SMO), vOICe, and self-motion + vOICe (vOICeSMO) conditions for the allocentric task are presented for a VI participant in the **top panels** and for a sighted participant in the **bottom panels.** The predicted estimate (MLE) was calculated for each subject, and then averaged, by entering the individual vOICe ($\sigma_{vOICe}$) and self-motion ($\sigma_{SMO}$) measure of variable error into the equation $\frac{\sigma_{\mathrm{vOICeSMO}\;=}^{2}\;\;\;\;\;\sigma_{vOICe}^{2}\sigma_{SMO}^{2}}{\;\sigma_{vOICe}^{2}+\sigma_{SMO}^{2}}$.

The individual estimates were obtained in the same way as described in Experiment 1. Figure 5 gives an example of variable and constant errors for a VI and a sighted participant.

#### Sighted Group

**Figure 6:**
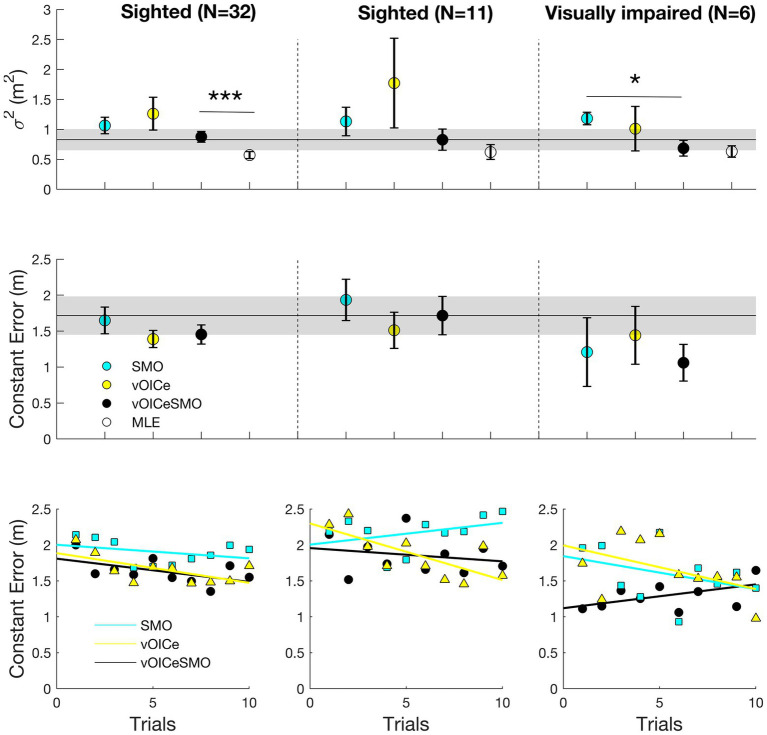
Average variable error (**top panels**), constant error (**middle panels**), and relation between average constant error and number of trials (**bottom panels**) for the allocentric task. Average results for the entire sighted group (*N* = 32; **left panels**), the sighted group that performed the task with the same order of the visually impaired (VI) group (*N* = 11; **middle panels**), and the VI group (*N* = 6; **right panels**). vOICe = vOICe condition alone; SMO = self-motion alone; vOICeSMO = self-motion + vOICe. The marker labeled MLE (in white) in the top panels refers to the reduction in variability predicted by the MLE model. How MLE was calculated is explained in the Figure 5 caption. Error bars represent the standard error of the mean, and the shaded dark line represent the combined measure (vOICeSMO) for the eleven sighted (**middle panels**) as a reference to both the combined conditions of the entire sighted group and of the VI group. ^***^ *p* < 0.005; ^*^ *p* < 0.05. Please see Supplementary Figure S5 for a boxplot with median and IQR measures.

**Figure 7:**
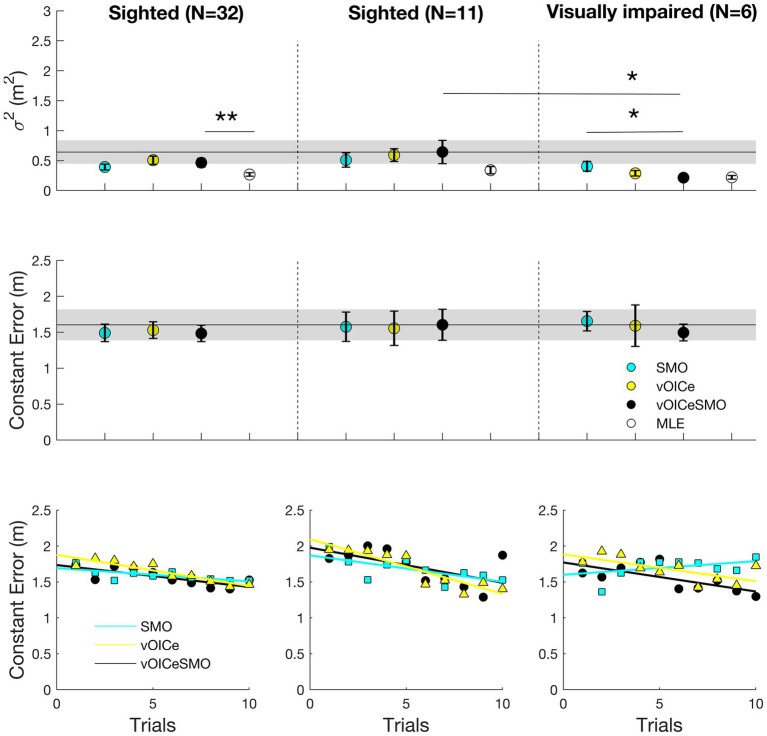
Average variable error (**top panels**), constant error (**middle panels**), and relation between average constant error and number of trials (**bottom panels**) for the egocentric task. Average results for the entire sighted group (*N* = 32; **left panels**), the sighted group that performed the task with the same order of the VI group (*N* = 11; **middle panels**), and the VI group (*N* = 6; **right panels**). vOICe = vOICe condition alone; SMO = self-motion alone; vOICeSMO = self-motion + vOICe. The marker labeled MLE (in white) in the top panels refers to the reduction in variability predicted by the MLE model. How MLE was calculated is explained in Figure 5 caption. Error bars represent the standard error of the mean, and the shaded dark line represent the combined measure (vOICeSMO) for the 11 sighted (**middle panels**) as a reference to both the combined conditions of the entire sighted group and of the VI group. ^**^ *p* < 0.01; ^*^ *p* < 0.05. Please see Supplementary Figure S6 for a boxplot with median and IQR measures.

Figures 6, 7 (left panels) shows the results for the allocentric and egocentric tasks, respectively. Wilcoxon tests were used to compare the variable error between the bimodal (vOICeSMO) and the unimodal conditions (vOICe and SMO) and between the measured bimodal (vOICeSMO) and the predicted bimodal (MLE, because estimated through the MLE model) conditions separately for the allocentric and egocentric tasks. The analysis showed no significant difference between vOICeSMO and the unimodal (vOICe and SMO) conditions for the allocentric task, *Z* ≤ −1.075, *p* ≥ 0.141, and one-tailed (despite a reduction in variable error for vOICeSMO when compared to the two unimodal conditions, see Figure 6 top left panel). There was, however, a significant difference between vOICeSMO and MLE (*Z* = −2.901, and *p* = 0.004), indicating that the reduction in variability for the bimodal condition was not accurately predicted by the MLE model. The same results were found for the egocentric task (vOICeSMO vs. vOICe and vOICeSMO vs. SMO: *Z* ≤ −0.777, *p* ≥ 0.218, and one-tailed; SSDMO vs. MLE: *Z* = −3.255, *p* = 0.001, see Figure 7 top left panel). A similar analysis was performed on the constant error measures (Figures 6, 7 left middle panel) and showed no significant difference between vOICeSMO and the unimodal conditions vOICe and SMO for both allocentric and egocentric tasks (allocentric: *Z* ≤ −1.085, *p* ≥ 0.139, and one-tailed; egocentric: *Z* ≤ −0.764, *p* ≥ 0.222, and one-tailed).

Finally, we examined whether sighted participants showed any learning effect across the 10 trials within each sensory condition (vOICe, SMO, and vOICeSMO) for allocentric and egocentric tasks separately. Pearson correlations were used to analyze whether the average constant error decreased with an increase in number of trials, i.e., whether participants’ accuracy increased with practice. For the allocentric task, as shown in Figure 6 bottom left panel, a significant learning effect was found for The vOICe condition (*r* = −0.627, *p* = 0.026, and one-tailed) and marginally for vOICeSMO (*r* = −0.547, *p* = 0.051, and one tailed), but not for SMO (*r* = −0.336, *p* = 0.171, and one-tailed). In addition, whereas vOICeSMO and vOICe also correlated positively (*r* = 0.669, *p* = 0.017, and one-tailed), vOICeSMO and SMO did not (*r* = 0.378, *p* = 0.140, and one-tailed), indicating that the increased in accuracy (or decrease in error) with trials in the bimodal condition (vOICeSMO) was driven by a learning effect and increased accuracy for The vOICe only condition.

For the egocentric task, as shown in Figure 7 bottom left panel, a significant learning effect was found for The vOICe (*r* = −0.916, *p* < 0.001, and one-tailed), the vOICeSMO (*r* = −0.761, *p* = 0.005, and one-tailed), and SMO (*r* = −0.717, *p* = 0.010, and one-tailed). Similar to the allocentric task, vOICeSMO and vOICe conditions also correlated positively (*r* = 0.745, *p* = 0.006, and one-tailed), while vOICeSMO and SMO did not (*r* = 0.528, *p* = 0.058, and one-tailed), indicating that in both tasks the increased accuracy in the bimodal condition (vOICeSMO) was driven by a learning effect and increased accuracy for The vOICe only condition (vOICe).

#### Sighted and Visually Impaired

Here, we report the results for the visual impaired group and for the sighted group that performed the two tasks with the same order of the visual impaired. We always tested the visual impaired group with the vOICeSMO (the combined condition) at the end while counterbalancing across participants the order of the other two conditions (vOICe and SMO). This was because of the small number of participants and because we were interested in examining whether any improvement with both sensory information (vOICe and self-motion) was possible for the VI in such a short session with The vOICe. Hence, below, after presenting the results for the VI group alone, we present the results for the 11 sighted participants that had the same task order as the visual impaired group and then compare these two groups’ performances.

#### Visually Impaired

Figures 6, 7 (right panels) shows the results for the allocentric and egocentric tasks for the six VI participants. Wilcoxon tests were used to compare the variable error between the bimodal (vOICeSMO) and the unimodal conditions (vOICe and SMO), and between the measured bimodal (vOICeSMO) and the predicted bimodal (MLEl) conditions separately for the allocentric and egocentric tasks. The analysis showed no significant difference between vOICeSMO and vOICe conditions for the allocentric task, *Z* = −1.363, *p* = 0.086, and one-tailed, while showing a significant difference between vOICeSMO and SMO (*Z* = −1.992, *p* = 0.023, and one-tailed). Additionally, no significant difference between vOICeSMO and MLE was found for this group (*Z* = −0.631, *p* = 0.528), indicating that the reduction in variability for the bimodal condition was well predicted by the MLE model. The results for the egocentric task returned a significant difference between vOICeSMO and vOICe, *Z* = −1.753, *p* = 0.04, and one-tailed, and between vOICeSMO and SMO, *Z* = −1.782, *p* = 0.037, and one-tailed, but no significant difference between vOICeSMO and MLE: *Z* = −0.315, *p* = 0.752 (see Figure 7 top right panel). This suggested that the VI group were able to reduce variability and improve their performance by integrating The vOICe information with self-motion as predicted by the MLE model. To examine whether the age of onset for the visual loss or the severity of the visual impairment (measure of visual acuity) correlated with the multisensory benefit shown by the VI group, we ran two linear regression analyses with the multisensory benefit as an outcome. We calculated the multisensory benefit as the difference in variable error between the combined condition (vOICe and self-motion) and the best unimodal condition (i.e., the condition that had the lower variable error between The VOICe alone or self-motion alone). Both regression analyses returned a non-significant result [age of onset: *F*(1,4) = 0.77, *p* = 0.795; visual acuity: *F*(1,3) = 0.506, *p* = 0.528].

A similar analysis was performed on the constant error measures (Figures 6, 7 right middle panel) and showed no significant difference between vOICeSMO and the unimodal conditions, vOICe and SMO, for both allocentric and egocentric tasks (*Z* ≤ −1.572, *p* ≥ 0.058, and one-tailed).

#### Sighted

Figures 6, 7 (middle panels) shows the results for the allocentric and egocentric tasks for the 11 sighted participants that performed the tasks with the same order of the VI (with vOICeSMO always at the end). Wilcoxon tests were used to compare the variable error between the bimodal (vOICeSMO) and the unimodal conditions (vOICe and SMO), and between the measured bimodal (vOICeSMO) and MLE conditions separately for the allocentric and egocentric tasks. The analysis showed no significant difference between vOICeSMO and the unimodal (vOICe and SMO) conditions for the allocentric task, *Z* ≤ −1.334, *p* ≥ 0.091, and one-tailed (despite a reduction in variable error for vOICeSMO when compared to the two unimodal conditions, see Figure 6 top middle panel). These results are equivalent to those of the entire sighted group. No significant difference between vOICeSMO and MLE was found (*Z* = −1.512, *p* = 0.130), although the MLE variable error was smaller than that measured for vOICeSMO. The same results were found for the egocentric task (vOICeSMO vs. vOICe and vOICeSMO vs. SMO: *Z* ≤ −0.578, *p* ≥ 0.281, and one-tailed; vOICeSMO vs. MLE: *Z* = −1.646, *p* = 0.100, see Figure 7 top middle panel). A similar analysis was performed on the constant error measures (Figures 6, 7 middle panel) and showed no significant difference between vOICeSMO and the unimodal conditions, vOICe and SMO, for both allocentric and egocentric tasks (allocentric: *Z* ≤ −1.156, *p* ≥ 0.124, and one-tailed; egocentric: *Z* ≤ −0.445, *p* ≥ 0.328, and one-tailed). These results again replicate those of the entire sighted group.

#### Comparing Visually Impaired and Sighted

Finally, we examined whether sighted participants and VI differed in any of the conditions (vOICe, SMO, and vOICeSMO) for the allocentric and egocentric tasks separately. For the variable error in the allocentric task, the two groups did not differ in all conditions (SMO: *U* = 25.000, *p* = 0.421; vOICe: *U* = 25.500, *p* = 0.451; vOICeSMO: *U* = 31.000, *p* = 0.841). For the variable error in the egocentric task, the two groups did not differ in the unimodal conditions (SMO: *U* = 28.000, *p* = 0.615; vOICe: *U* = 15.000, *p* = 0.070) but did differ in the combined condition (vOICeSMO: *U* = 13.000, *p* = 0.044) with VI performing better than sighted when having both cues (Figure 7, top middle and right panels). No significant differences were found for the constant error when comparing the two groups on all conditions (*U* ≥ 17.000, *p* ≥ 0.108, see Figures 6, 7 top middle and right panels).

Finally, we examined the difference in learning (decreased accuracy) with the number of trials between sighted and VI by running multiple linear regressions with group and trials as predictors and constant error (accuracy) for each condition as outcome. For the allocentric task (Figure 6 bottom middle and right panels) in the SMO condition, we found a significant regression equation [*F*(2,19) = 7.957, *p* = 0.004], with an R-square of 0.484. Of the two predictors only “group” contributed to the significant result found (*p* = 0.001). For The vOICe, we also found a significant regression equation [*F*(2,19) = 5.596, *p* = 0.014], with an R-square of 0.397; however, this time only the “trials” predictor contributed to this significant effect (*p* = 0.009). Finally, for the vOICeSMO, we found a similar result to SMO, in that we found a significant regression equation [*F*(2,19) = 13.805, *p* < 0.001], with an R-square of 0.619 with “group” as only contributor (*p* < 0.001).

For the egocentric task (Figure 7 bottom middle and right panels) in the SMO condition, we did not find a significant regression equation [*F*(2,19) = 0.413, *p* = 0.668], with an R-square of 0.046. For The vOICe, similarly to the allocentric task, we found a significant regression equation [*F*(2,19) = 14.855, *p* < 0.001], with an R-square of 0.636, with only the “trials” predictor contributing to this significant effect (*p* < 0.001). Finally, we found a significant regression equation for vOICeSMO [*F*(2,19) = 7.836, *p* = 0.004], with an R-square of 0.480 and both “trials” (*p* = 0.004) and “group” (although only marginally: *p* = 0.051) contributing to this effect.

## Discussion

We set out to explore how well two non-visual senses can be integrated to provide a representation of space that aids navigation in egocentric or allocentric tasks. First, in Experiment 1, we examined in a group of sighted individuals whether using one SSD (The vOICe providing an auditory display and the BrainPort providing a tactile display) or both simultaneously provided better performance in transferring information from a map to a real, 3D space. Both the egocentric and allocentric tasks revealed that the variable error did not reduce in the combined condition and was not well explained by the MLE model (e.g., Ernst and Banks, 2002). Performance for The vOICe on its own was less biased compared to the combined condition for the more difficult allocentric task. Consistent with this, there was no learning effect across trials for The vOICe as there was for the BrainPort. In fact, the constant error for The vOICe starts at a lower value and converges with the other by the end of the 10 trials; in contrast, for the egocentric trials, the constant errors were equivalent at the start and followed a uniform decrease in error across all conditions by the end of the 10 trials. A learning effect occurred for both The vOICe and BrainPort in the egocentric task but not in the combined condition. The lack of multisensory benefit in both tasks and the lack of learning effect for the combined condition in the allocentric task could be a consequence of task difficulty and sensory overload. It is plausible to conclude that learning to use two new devices and their delivered sensory information requires higher sensory and cognitive load than when only one device is used. As learning to use the two devices and then benefitting from their integration may require more time and training, it may be possible that a longer period of learning and use of the two devices together would allow for the multisensory benefit to emerge. An alternative explanation for the lack of multisensory integration as found in Experiment 1 is that the information provided by the two devices is too different (e.g., The vOICe transforms visual images by scanning sequentially from left-to-right while the BrainPort transforms visual information into a pattern of simultaneous electrical stimulation). This mismatch in received information may tell the brain that these cues probably belong to separate events, thus impeding integration. Future studies could test both possible explanations by training participants for a longer time when two or more devices are used and by trying to match the type of information provided by the different devices more closely. To this end, the use of new and improved sensory substitution systems such as the sound of vision (SoV; Caraiman et al., 2017) that uses depth cameras to provide the users with rich tactile and/or auditory information would be optimal. Using a system like SoV would allow for a closer match of tactile and sound information when forming a multisensory spatial representation, thus allowing for a better assessment of multisensory benefit during navigation in blind users. Also using SoV will also help overcome some of the limitations of the SSDs used here, for example, by avoiding the constraints of a tongue display which can limit the user’s speech and consequently imped social interaction in real life situations.

In Experiment 2, we examined whether a group of sighted and a group of VI individuals could integrate two senses in a navigation task, namely self-motion and the auditory SSD (The vOICe) in an egocentric and an allocentric spatial navigation task. For the sighted group, there was no improvement in the combined condition compared to the unimodal conditions. The sighted showed a decrease in constant error across trials with The vOICe and the combined condition in both allocentric and egocentric tasks, and they had an increase in accuracy for SMO in the egocentric task. Interestingly, improvement in the combined condition correlated with that in The vOICe condition, suggesting that the performance with The vOICe was driving the improvement in the combined condition. Given a longer period of learning, perhaps this could have resulted in a significant reduction in variability in the allocentric task. However, the variable error in the egocentric task was suggestive of good performance and thus indicative of a ceiling effect that might have limited any improvement.

The VI participants in Experiment 2 had a reduction in variability for the combined condition in both egocentric and allocentric tasks; the combination of The vOICe and self-motion was significantly different than self-motion alone in the allocentric task, and the combination was better than both unimodal conditions in the egocentric task. The MLE model predictions did not differ from the combined condition; this indicates that the MLE model predicted performance well and that the VI did benefit from combining The vOICe auditory display and self-motion into a multisensory representation even with such a short period of training and testing.

The multiple regression analyses provided converging evidence that both allocentric and egocentric tasks showed a difference in group performance across trials for the combined condition, such that there was a learning effect. For both tasks, there were no group differences in The vOICe only condition, in that both sighted and VI improved with number of trials. In the allocentric task, though, the group effect was driven by the fact that the VI had a starting error that was much lower for the combined condition than the unimodal condition, unlike the sighted. In contrast, the egocentric task resulted in different performance between the VI and the sighted, with both groups showing improvement but the VI showing greater improvement. Finally, the two groups differed for self-motion in the allocentric task because the VI improved their performance with the number of trials while the sighed did not. Hence, the VI seem to be able to benefit more from the non-visual multisensory representation of space and even self-motion alone. However, in the egocentric task, VI did not improve in the self-motion condition, while sighted did. Finally, in the allocentric task the combined condition for the VI was already better (had lower constant error) than the unimodal conditions from the start (the first trial).

A significant quantity of research and development has been dedicated to The vOICe, demonstrating it allows successful object recognition and localization (Auvray et al., 2007) and offers superior spatial resolution in comparison to other SSDs (Proulx et al., 2014b). However, there is a comparative paucity of research demonstrating the efficacy of The vOICe in the context of spatial navigation. In contrast, the BrainPort has been demonstrated to convey inferior spatial resolution, but superior temporal resolution to The vOICe (Bach-y-Rita and Kercel, 2003), and (perhaps as a result of this) has demonstrable success in assisting VI navigation (Chebat et al., 2011, 2015). Nevertheless, depth perception remains a critical stumbling block for both devices. The first experiment removed the lack of depth perception as a limiting factor for navigation performance by using aerial maps; however, the results indicated that such information was better encoded and utilized when delivered by one device alone rather than both in combination.

Why might these devices not show evidence of optimal integration? Ernst and Banks (2002) Bayesian integration model suggests that optimal integration is better achieved when multiple sensory inputs have similar reliability; additionally, the Convergent Active Processing in Inter-Related Networks (CAPIN; Schinazi et al., 2016) theory postulates that in the blind in absence of the visual modality, other cues receive greater weights than they would have if vision was available (Millar, 1994). Hence, CAPIN postulates that in blindfolded sighted individuals the weights remain unchanged with vision receiving more weight than the other remaining cues. Hence, the lack of multisensory benefit in blindfolded sighted individuals in both experiments could be attributed to this inability to reweight the non-visual information based on the temporarily lack of vision. Furthermore, while audition contributes to a pictorial concept of space, Millar (1994) suggests that haptics exert the greater influence; therefore, information delivered *via* the auditory modality using The vOICe may have been attributed lower reliability than that from the BrainPort, preventing integration. However, our results for the variable error in Experiment 1 show that this explanation is unlikely as the two devices allowed for the same level of reliability, and further, The vOICe did have a lower constant error when compared to the BrainPort. Hence, we believe that the length of training/learning (10 repetitions for conditions) was just not enough to result in an integrated spatial representation using the two devices.

It was striking that, in Experiment 2, learning object locations through The vOICe provided similar precision (variable error) in navigation as self-motion, particularly considering the relatively short training (1 h) participants received. Results for the sighted individuals indicated no significant benefit in navigation precision in the combined condition, in neither type of spatial representation. This is in line with a body of research which suggested that cue competition, rather than integration, may occur in navigation tasks when a level of discrepancy is perceived among the cues (Tcheang et al., 2011; Garcia et al., 2015; Petrini et al., 2016). That is, although sighted individuals could use both cues in isolation with a similar level of precision, they probably discard the information afforded by The vOICe (which is less familiar) and relied on self-motion (given the high level of familiarity and that self-motion is what they were using to walk to the target object).

A significant difference was found between allocentric and egocentric variable error values, with egocentric navigation being more precise. This effect was expected, and in line with previous literature (e.g., Pasqualotto et al., 2013; Adame et al., 2014; Iachini et al., 2014), because in the allocentric navigation participants had to estimate two distances and infer the turning angle between two objects, unlike in egocentric navigation where they estimated one distance in a straight line. Moreover, this difference is indicative of the efficiency of the current design in testing both egocentric and allocentric spatial representations. This is the first study to test The vOICe in navigation tasks, previous research only assessing object recognition or object-locating tasks (Poirier et al., 2007; Proulx and Harder, 2008; Ptito et al., 2008). Even more, to our knowledge no study up to date has investigated in a controlled environment the efficiency of The vOICe in comparison to self-motion. Arguably our participants had much more extensive experience in navigating with SMO (walking in darkness), as opposed to navigating with The vOICe. Furthermore, studies using The vOICe have employed significantly more extensive training, with Auvray et al. (2007) providing 15 h, Amedi et al. (2007) providing 40 h of directed training, and Ward and Meijer (2010) examining its use after several months of usage. The current study thus provides a strong evidence that The vOICe can be effective even with a very short period of training. Although, this result is novel in showing the efficiency of The vOICe for navigation with minimal training, it adds to the existing evidence showing that several SSDs (e.g., EyeCane and tongue unit displays) can aid spatial navigation with a short period of training ranging from few minutes to few hours (Chebat et al., 2011, 2015; Maidenbaum et al., 2014; Kolarik et al., 2017). Moreover, the present findings demonstrate that participants are able to transfer spatial information gathered with The vOICe into self-motion information, since in one condition the spatial representation of objects was learned with The vOICe but the recall was tested with self-motion alone. This supports the idea that participants tend to feed in information acquired through any modality into a multisensory cognitive map pertaining spatial representations of the environment (Tcheang et al., 2011; Schinazi et al., 2016), which they can subsequently use in navigation.

The improvement in performance with number of trials when using The vOICe alone can be indicative of increased decoding abilities in participants. This assumption is supported by studies showing that after extensive training, visual-to-audio sensory substitution can determine instantaneous visual images of the scanned environment (Auvray et al., 2007; Ward and Meijer, 2010; Kim and Zatorre, 2011). In other words, users of SSDs can shift from effortful processing of the new sound information to automatically creating visual images by listening to the soundscapes (Brown et al., 2011). It is known that as high effort processing of navigation cues shifts to automatic processing, the pressure on cognitive resources also decreases (Loomis et al., 2002; Klatzky et al., 2006; Picinali et al., 2014). Therefore, cognitive resources required for processing each modality could decrease with practice, which leaves scope for integration. This is especially relevant for the more complex allocentric task, since decoding more complex sensory information equates to a higher cognitive load (Klatzky et al., 2006). To assess whether optimal integration of self-motion and The vOICe information can be achieved in sighted individuals with further practice, future studies could use the task presented here but with a higher number of trials.

Although a multisensory representation of space and the use of the VOICe and self-motion together did not result in a benefit for the sighted individuals, it did for the VI group. The VI group was able to reduce their variability and increase their precision when using The vOICe and self-motion together despite the very short period of training and low number of repetitions for each sensory condition. The use of a non-visual multimodal representation of space by blind people (Schinazi et al., 2016) is consistent with evidence from neuroscience, which suggests the brain does not process sensory information rigidly but re-organizes when a sensory ability is lost (De Volder et al., 1999; Poirier et al., 2007; Kupers et al., 2011). Our results are also in line with recent findings that show how using an audiotactile map to navigate the environment is more efficient for blind individuals than a tactile map and only walking (Papadopoulos et al., 2018). Hence, the present findings demonstrate that VI and blind persons can optimally integrate (Ernst and Banks, 2002) the new information coming from The vOICe with the available information from self-motion into a richer multisensory cognitive map than when using only self-motion. This brings support to the convergent model of spatial learning (Schinazi et al., 2016) in the blind and VI, by showing that even when using less effective (when compared to vision) cues for navigation, blind and VI can learn to perform as well as sighted by increasing their precision through non-visual multisensory integration.

A limitation to the generalization of the findings reported here, however, is the small sample size of VI individuals, and its heterogonous composition (i.e., different onset of visual impairment or blindness and severity of the condition). These limitations are not uncommon (e.g., Gaunet and Thinus-Blanc, 1996; Kupers et al., 2010, 2011; Gagnon et al., 2012; Chebat et al., 2015; Garcia et al., 2015; Kolarik et al., 2017) and the decision to include participants with different types of visual impairment was driven by the necessity to determine the level of generalization of our findings. That is, we wanted to examine whether different types of visual impairments could benefit from using The vOICe with self-motion when navigating to a target location. Additionally, the onset and severity of the visual impairment did not correlate with the benefit achieved by the participants in the combined condition, and overall, the variability among visual impaired participants’ performances was low. Hence, our findings do show that integrating The vOICe information with self-motion during both egocentric and allocentric navigation can benefit persons with different durations and types of visual loss. Another limitation of the present study is the use of a controlled and relatively simple navigation task. Although our findings are promising they require further testing in the complex world outside the laboratory setting or alternatively by using virtual reality environments to simulate complex real situations (e.g., Chebat et al., 2015; Caraiman et al., 2017). In line with this limitation, it would be good for future studies to also obtain measures of performance time (Caraiman et al., 2017) in addition to error and variance to examine whether the availability of two cues together can rapidly speed up the navigation and way finding task. Hence, further studies will be able to assess if the found multisensory benefit in VI persons shown here can extend to daily life tasks and situations and to speed of performance.

Drawing on these findings, the applicability of The vOICe during navigation for the blind and VI population seems very promising. Firstly, the current study showed that both egocentric and allocentric information can be learned by using The vOICe soundscapes to form a rich cognitive map that can subsequently be used to navigate the environment. Moreover, it shows that VI and blind individuals can learn to integrate The vOICe soundscapes and self-motion more readily than sighted, because they usually outperform sighted individuals when using either of these cues during spatial representation encoding and navigation tasks (Tinti et al., 2006; Schinazi et al., 2016). This means that the benefit of using The vOICe alone and in combination with self-motion during spatial navigation can be achieved rapidly in VI and blind individuals with minimum training, hence removing one of the main barriers for the adoption of these SSDs in everyday life. This novel finding is promising in defining a new way to aid the blind population and further our understanding of spatial cognition after sensory loss. In fact, our results highlight how exploiting non-visual multisensory integration to develop new assistive technologies could be key to help the blind and VI persons especially due to their difficulty in attaining allocentric information (Pasqualotto et al., 2013; Schinazi et al., 2016).

## Data Availability Statement

The datasets generated for this study are available on request to the corresponding author.

## Ethics Statement

The studies involving human participants were reviewed and approved by the Psychology Research Ethics Committee (PREC), University of Bath. The patients/participants provided their written informed consent to participate in this study.

## Author Contributions

KP, MP, TL-E, EO'N, SL-S, and CJ developed the study idea and the study design. CJ, TL-E, MS, and SL-S recruited and tested participants. KP, SL-S, and CJ analyzed the data. All authors contributed to the writing of the paper.

## Conflict of Interest

The authors declare that the research was conducted in the absence of any commercial or financial relationships that could be construed as a potential conflict of interest.

## Supplementary Material

The Supplementary Material for this article can be found online at: https://www.frontiersin.org/articles/10.3389/fpsyg.2020.01443/full#supplementary-material.
