# Supplementary material for: Efficiency of Sensory Substitution Devices Alone and in Combination With Self-Motion for Spatial Navigation in Sighted and Visually Impaired
Source: Front Psychol. 2020 Jul 10;11:1443. doi: 10.3389/fpsyg.2020.01443 (PMC7381305; doi:10.3389/fpsyg.2020.01443)
Supplement: Supplementary file 1 [file Data_Sheet_1.PDF]

Efficiency of sensory substitution devices alone and in combination with self-motion for spatial navigation in sighted and visually impaired

Crescent Jicol*[1], Tayfun Lloyd-Esenkaya*[2], Michael Proulx[1], Simon Lange-Smith, Meike Scheller[1], Eamonn O'Neill[2], Karin Petrini[1]

[1] Department of Psychology, University of Bath, UK

[2] Department of Computer Science, University of Bath, UK

[3] Liverpool John Moores University, UK

*CJ and TLE share first authorship

Corresponding Author:

Dr Karin Petrini

Senior Lecturer/Associate Professor

Department of Psychology

University of Bath

Claverton Down

Bath

BA2 7AY

United Kingdom

Email: K.Petrini@bath.ac.uk

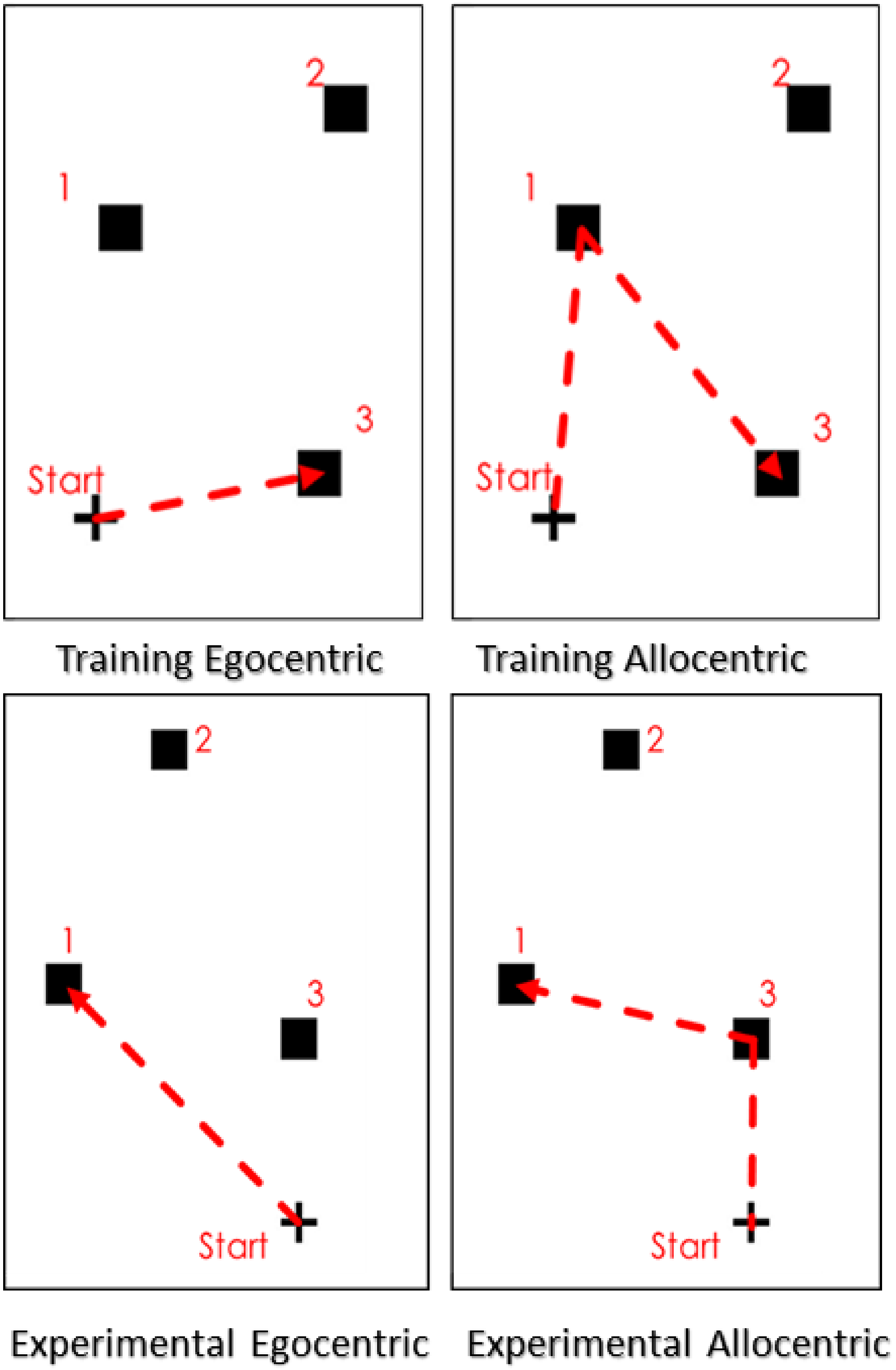

Figure S1. The configurations for the target points for the training and experimental conditions and for egocentric and allocentric navigation. The cross represents the start point and the three numbered squares represent the three objects that were utilised. The dotted red lines represent the trajectory of the navigation that was completed by participants in each type of trial.

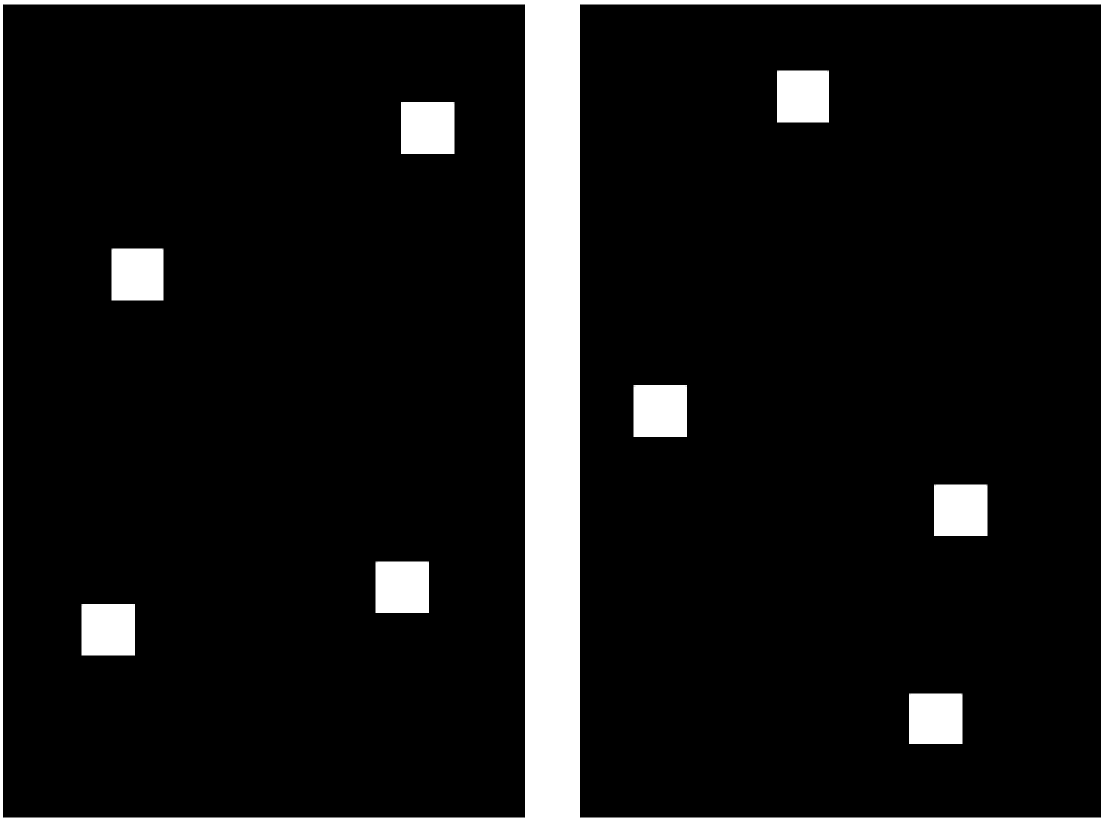

Figure S2. The ‘aerial maps’ that were used to train participants with the object configuration in the training and experimental trials. The four white squares represent the start point and the three objects.

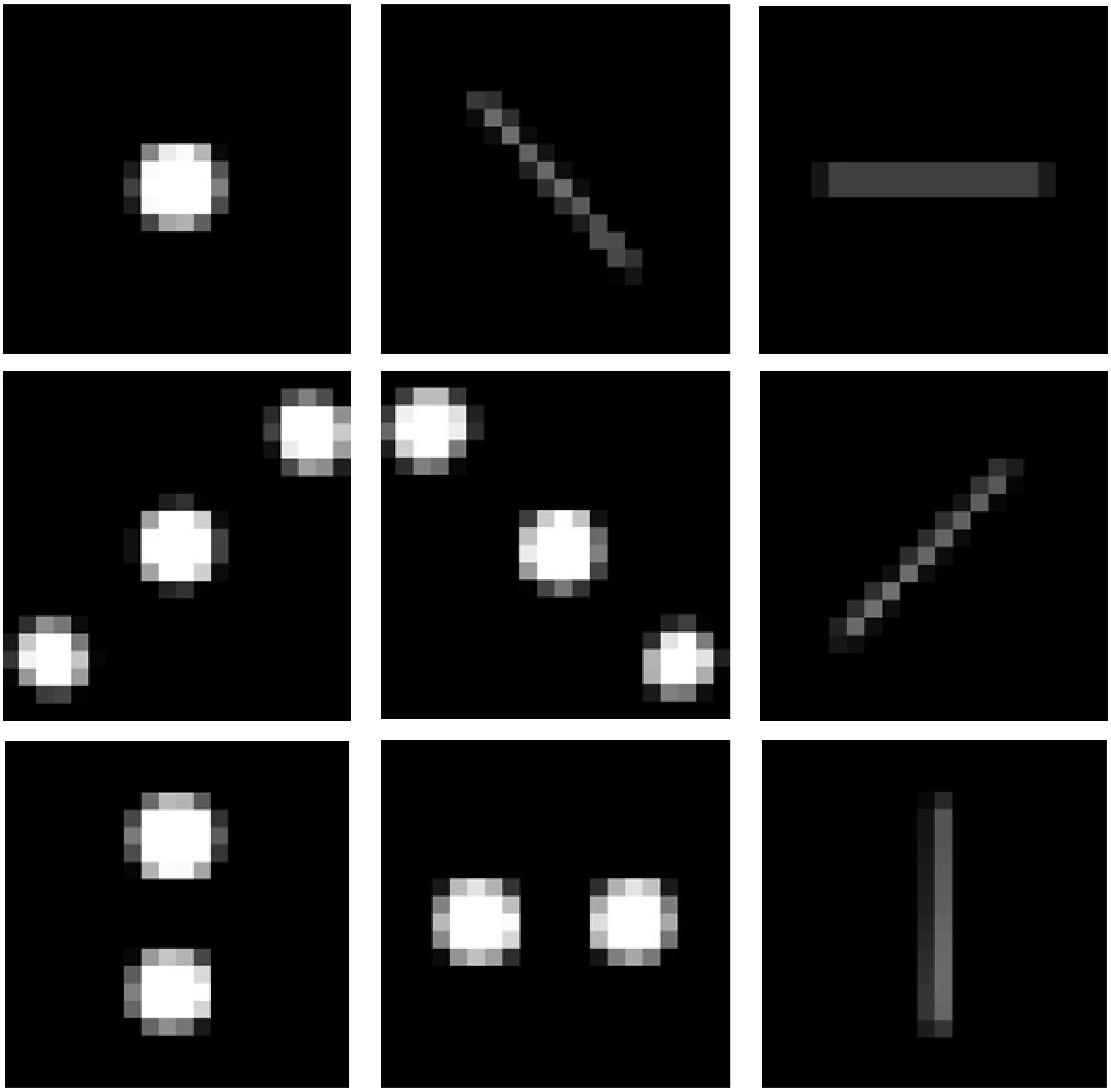

Figure S3. The training stimuli that were used for training with The vOICe and the TDU. These constituted of ascending, descending, horizontal and vertical lines or arrangements of dots in order to familiarise the participants with how the two devices encoded such information.

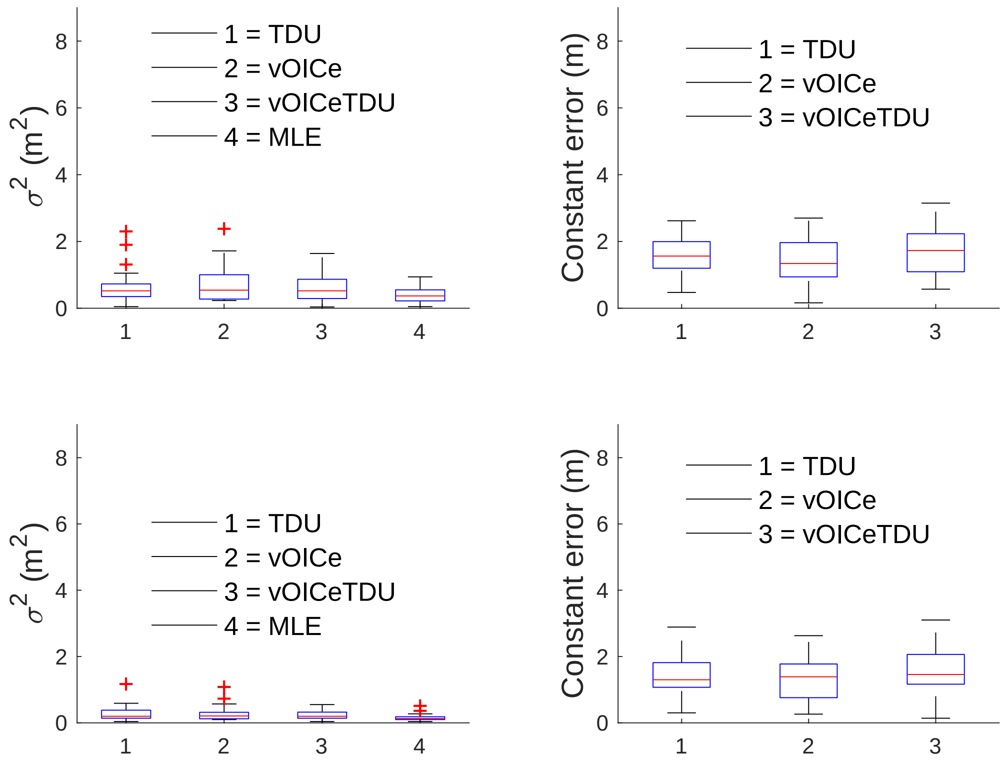

Figure S4. Average variable error (left panels), constant error (right panels) boxplots for the allocentric task (top panels) and egocentric task (bottom panels) in Experiment 1. vOICe = vOICe condition alone; TDU = tongue display unit/BrainPort alone; vOICeTDU = vOICe + tongue display unit/BrainPort. The marker labelled MLE in the left panels refers to the reduction in variability predicted by the maximum likelihood estimation model.

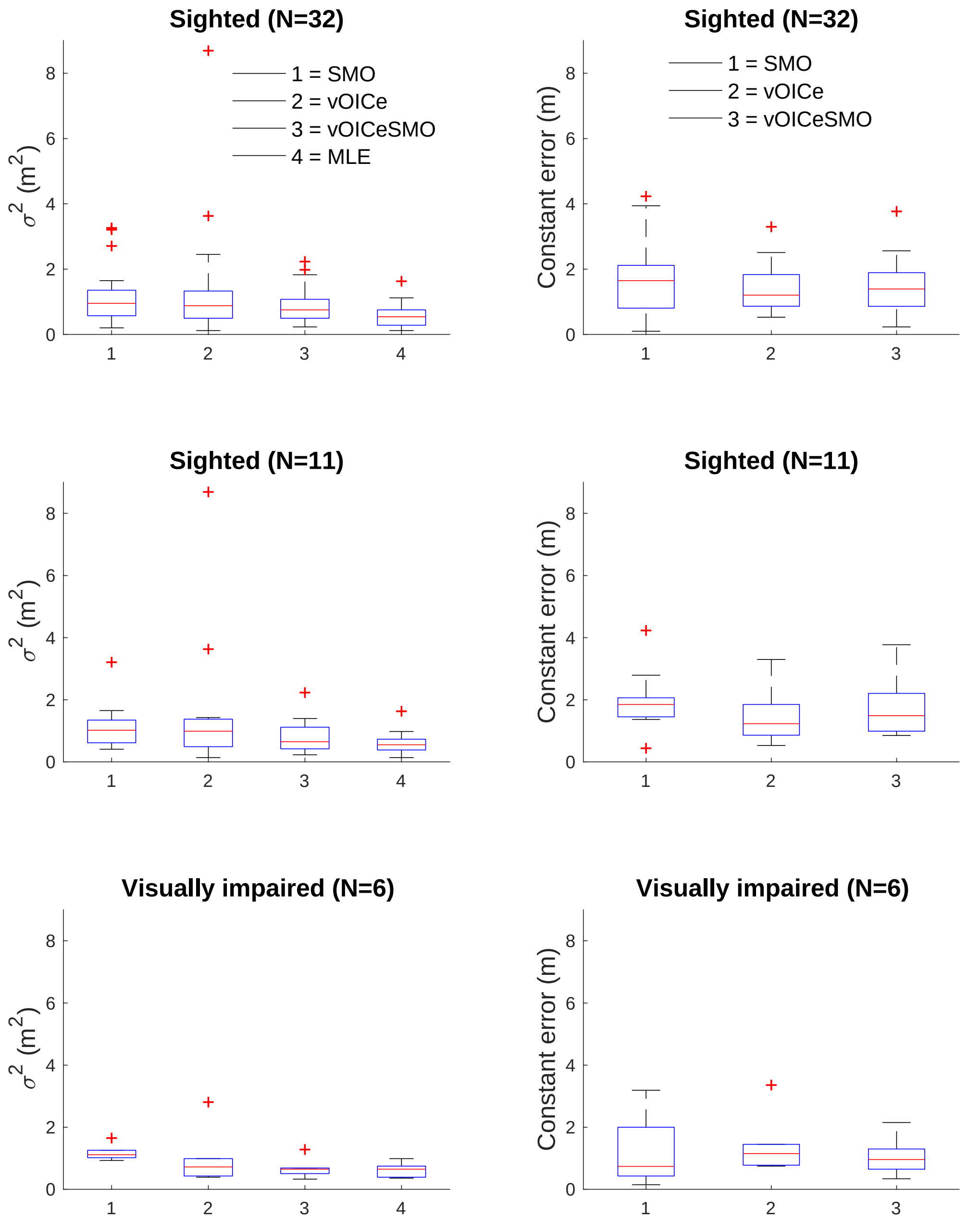

Figure S5. Average variable error (left panels), constant error (right panels) boxplots for the allocentric task in Experiment 2. Results for the entire sighted group (N=32; left panels), the sighted group that performed the task with the same order of the visually impaired group (N=11; middle panels), and the visually impaired group (N=6; right panels). vOICe = vOICe condition alone; SMO = self-motion alone; vOICeSMO = self-motion + vOICe. The marker labelled MLE (in white) in the top panels refers to the reduction in variability predicted by the maximum likelihood estimation model.

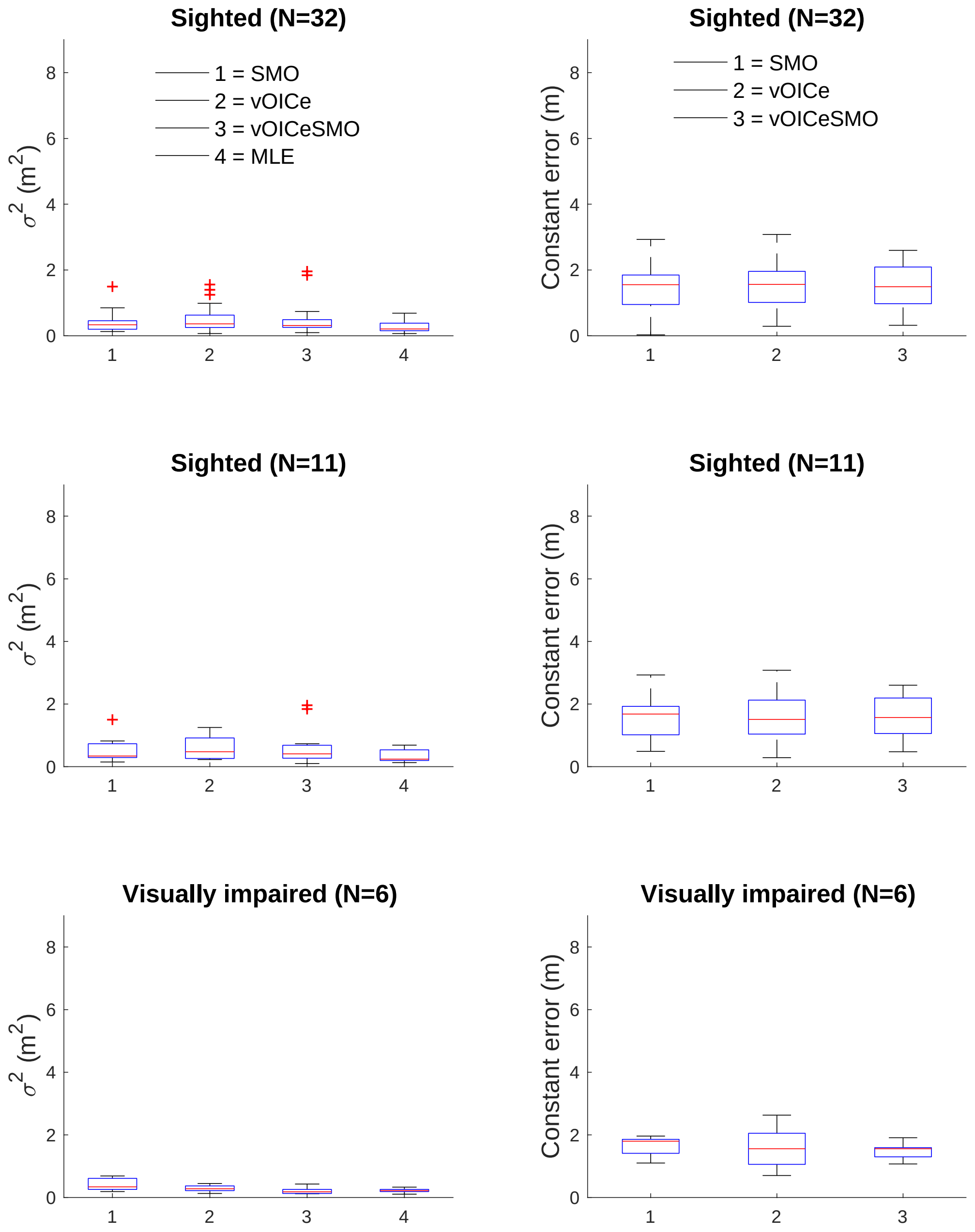

Figure S6. Average variable error (left panels), constant error (right panels) boxplots for the egocentric task in Experiment 2. Results for the entire sighted group (N=32; left panels), the sighted group that performed the task with the same order of the visually impaired group (N=11; middle panels), and the visually impaired group (N=6; right panels). vOICe = vOICe condition alone; SMO = self-motion alone; vOICeSMO = self-motion + vOICe. The marker labelled MLE (in white) in the top panels refers to the reduction in variability predicted by the maximum likelihood estimation model.
